# Increased expression of Fibrinogen-Like Protein 2 is associated with poor prognosis in patients with clear cell renal cell carcinoma

**DOI:** 10.1038/s41598-017-13149-x

**Published:** 2017-10-04

**Authors:** Ming Tang, Xu Cao, Peng Li, Kun Zhang, You Li, Quan-you Zheng, Gui-qing Li, Jian Chen, Gui-lian Xu, Ke-qin Zhang

**Affiliations:** 1 Department of Nephrology, Southwest Hospital, Third Military Medical University, Chongqing, 400038 China; 2 Department of Immunology, Third Military Medical University, Chongqing, 400038 China

## Abstract

Fibrinogen-like protein 2 (FGL2) is highly expressed in various tumour tissues and plays a vital role in tumour initiation and progression. This study evaluated the clinical significance of FGL2 in patients with clear cell renal cell carcinoma (ccRCC). FGL2 expression in fresh and 170 archived paraffin-embedded ccRCC tissues was measured by quantitative RT-PCR, western blotting, and immunohistochemitry. FGL2 expression was significantly upregulated in ccRCC. Statistical analyses by using Kaplan–Meier method showed that high FGL2 expression was associated with poor overall survival (OS) and recurrence-free survival (RFS) of patients with ccRCC. Multivariate analyses indicated that FGL2 was as an independent prognostic factor of survivaland that tumoural FGL2 levels could significantly predict the prognosis of patients with early-stage ccRCC. Nomogram systems, which integrated FGL2 expression and other clinical parameters, were established and were found to be better than TNM staging in predicting the OS and RFS of patients with ccRCC. FGL2 silencing led to a significant reduction in cells viability and increase in cells apoptosis, accompanied with a reduced ERK1/2 and p38 MAPK activation, in ccRCC cells. Thus, our results suggest that high FGL2 expression is a novel, independent, and an adverse prognostic factor of clinical outcomes in patients with ccRCC.

## Introduction

Kidney cancer is the third most common urological cancer and accounts for approximately 3% of all adult malignancies^1^. Renal cell carcinoma (RCC) is the most common renal tumour; it arises from the proximal tubular epithelium and accounts for approximately 90–95% of all cases of renal tumours^2^. RCC is often asymptotic, and approximately 30% patients with RCC are diagnosed at the metastatic stage because of the non-availability of good prediction methods^3^. RCC comprises many histological subtypes; however, clear cell RCC (ccRCC), which accounts for 75–80% of all primary kidney malignancies, is the most common RCC subtype^4^. Recently, some predicting models have been investigated to evaluate the risk of ccRCC and TNM staging and Fuhrman grade are widely used systems^5^. Stage, size, grade, and necrosis (SSIGN) score and University of California Integrated Staging System (UCISS) are also commonly used^6^. However, these systems cannot accurately predict the risk of ccRCC. Therefore, it is important to identify a ccRCC marker for predicting the prognosis of patients with ccRCC.

Fibrinogen-like protein 2 (FGL2; also called FGL2 prothrombinase), which was cloned from cytotoxic T lymphocytes and showed 36% homology to fibrinogen β and γ chains, is a member of fibrinogen family of proteins^7,8^. FGL2 has prothrombinase activity and performs potent immunoregulatory functions during hepatitis, allograft rejection, and abortion^7,9,10^. FGL2 expression is obviously upregulated in different cancers such as liver, colon, breast, and lung cancers^9^. A recent study reported that FGL2 promotes angiogenesis and tumorigenesis in prostate cancer through FGF-2/ERK signalling^11^. Moreover, FGL2 promotes the growth and angiogenesis of human hepatocellular carcinoma (HCC) cells in mouse xenograft injected models^12,13^. FGL2 activity increases in B-cell lymphomas but decreases after remission; moreover, detection of FGL2 activity in peripheral blood mononuclear cells (PBMCs) could be a biomarker for B-cell lymphomas^14^. However, limited studies have investigated the relationship between FGL2 expression and ccRCC development and no study has focused on the prognostic function of FGL2 in patients with ccRCC after partial or total nephrectomy.

In the present study, we investigated the association of FGL2 expression with the clinicopathological features, overall survival (OS), and recurrence-free survival (RFS) of patients with ccRCC and established novel nomogram systems that integrated FGL2 expression and other clinical parameters to predict the OS and RFS of patients with ccRCC. In addition, the possible mechanism of the role of FGL2 in ccRCC was further investigated *in vitro* cell cultures.

## Results

### FGL2 overexpression in ccRCC specimens

**Figure 1:**
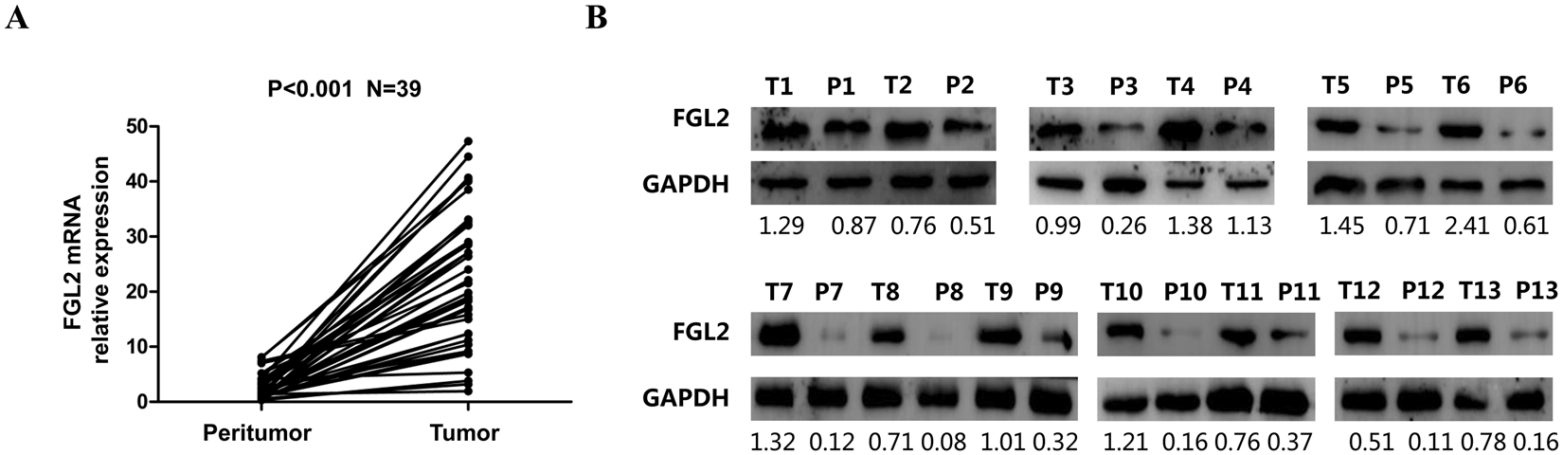
FGL2 overexpression in ccRCC specimens. (**A**) FGL2 mRNA expression were determined by qRT-PCR in 39 paired fresh tumoural tissues and peritumoural tissues, respectively. GAPDH was used as control. Paired-samples t test, *P* < 0.05 was regarded as statistically significant. (**B**) The protein expression levels of FGL2 in 13 paired tumoural tissues and peritumoural tissues were evaluated by Western blotting assay. GAPDH was used as a loading control. P = Peritumour; T = Tumour (The blots were cropped and full-length blots were presented in Fig. S1).

FGL2 expression in ccRCC tissues was assessed by detecting FGL2 mRNA expression in 39 paired fresh tumoural tissues and peritumoural tissues by performing qRT-PCR and the basic information of patients were presented in Supplementary Table S1. FGL2 mRNA expression was significantly higher in tumoural tissues than in peritumoural tissues (*P* < 0.001; Fig. 1A). FGL2 protein expression was measured by performing western blotting of tumoural and peritumoural tissues. Result of western blotting was consistent with that of qRT-PCR (Fig. 1B). These results indicate that FGL2 expression is significantly upregulated in fresh ccRCC tissues.

### Immunohistochemical evaluation and association between FGL2 expression and clinicopathological parameters of patients with ccRCC

**Figure 2:**
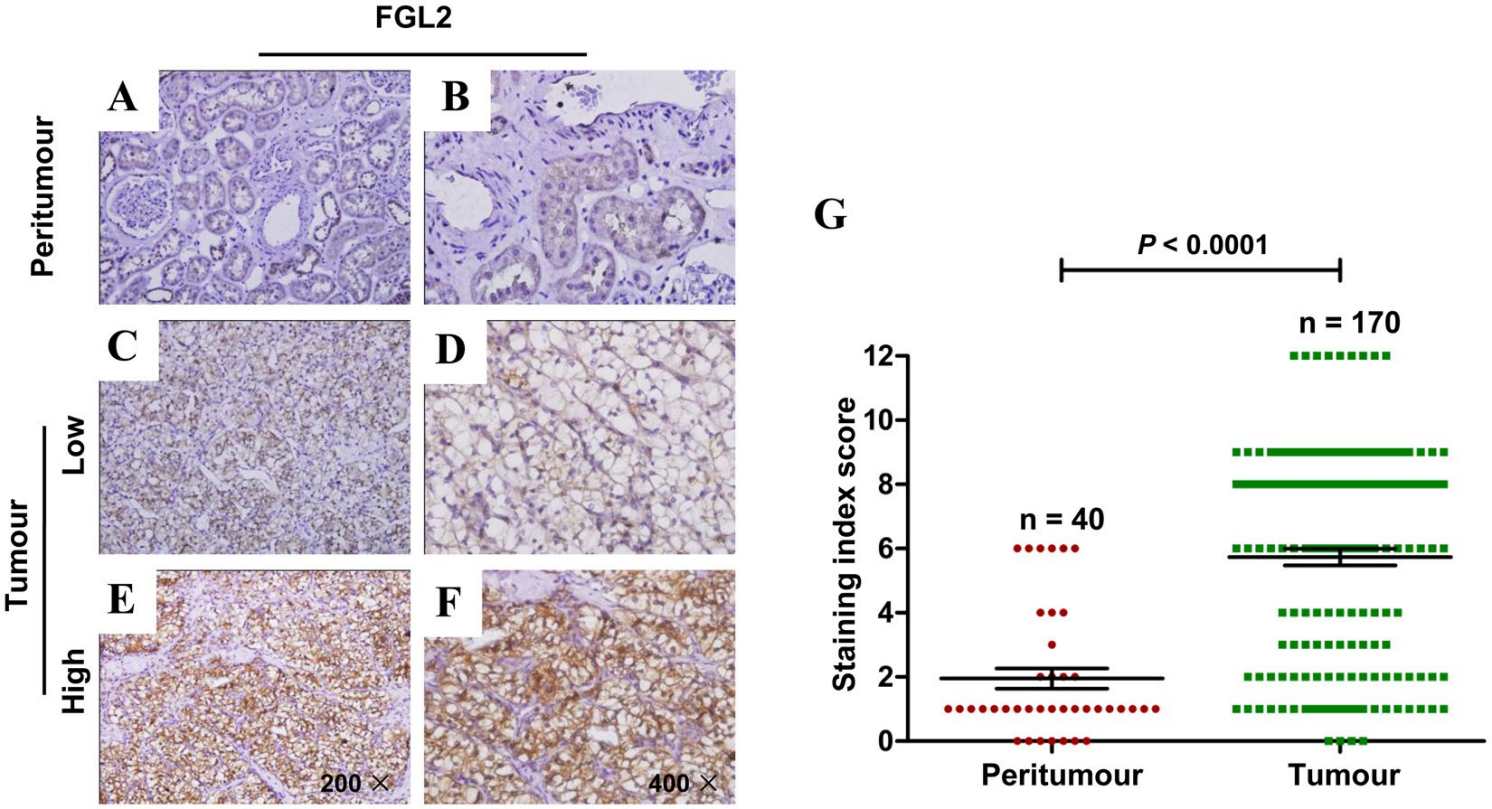
The expression of FGL2 in ccRCC tissues based on immunohistochemistry staining. (**A** and **B**) FGL2 expression was detected by IHC in peritumoural tissue.(**A**, original magnification ×200 and B, original magnification ×400). (**C** and **D**) Low FGL2 expression in tumoural tissues. (**C**, original magnification ×200 and **D**, original magnification ×400). (E and F) High FGL2 expression in tumoural tissues. (**E**, original magnification ×200 and **F**, original magnification ×400). (**G**) Staining index score of FGL2 expression in tumoural tissues and peritumoural tissues. *P*-value, calculated by Mann Whitney test, <0.05 was regarded as statistically significant.

Next, we evaluated FGL2 expression pattern in 170 paraffin-embedded ccRCC tumour tissues and 40 peritumoural renal tissues by performing immunohistochemical analysis. Immunohistochemical analysis showed that FGL2 expression was mainly localized in the membrane of ccRCC cells (Fig. 2A–F), and that staining index scores were different for different specimens (Fig. 2G). However, no or weak FGL2 staining was observed in peritumoural tissues (Fig. 2A,B). According to the FGL2 staining index score, 67 (39.4%) tumoural specimens and 103 (60.6%) tumoural specimens showed low (Fig. 2C,D) and high (Fig. 2E,F) FGL2 expression, respectively.

Patients showing high FGL2 expression had significantly large tumours (*P* = 0.002), high T classification (*P* = 0.002), and high TNM stage (*P* = 0.003) (Table 1). No significant association was observed between FGL2 expression and other clinicopathological parameters such as patient age, gender, Fuhrman grade, necrosis, and N classification.

**Table 1:** Correlation between FGL2 expression and clinicopathologic characteristics of ccRCC patients.

Characteristic	Cases	FGL2 expression		*P* ^*b*^
	n = 170	Low(n = 67)	High(n = 103)	
Gender				0.997
Male	104 (61.2%)	41	63	
Female	66 (38.8%)	26	40	
Age(years)^a^				0.225
≤55	96 (56.5%)	34	62	
>55	74 (43.5%)	33	41	
Tunor size(cm)^a^				0.002*
≤4	89 (52.4%)	45	44	
>4	81(47.6%)	22	59	
T classification				0.003*
T1-T2	132 (77.6%)	60	72	
T3-T4	38 (22.4%)	7	31	
N classification				0.266
N0	162 (95.3%)	62	100	
N1	8 (4.7%)	5	3	
TNM stage				0.002*
I-II	138 (81.2%)	62	76	
III-IV	32 (18.8%)	5	27	
Fuhrman grade				0.763
1-2	117 (68.8%)	47	70	
3-4	53 (31.2%)	20	33	
Necrosis				0.714
Absent	153 (90%)	61	92	
Present	17 (10%)	6	11	

Abbreviations: FGL2 = Fibrinogen-like protein 2; ccRCC = clear cell renal cell carcinoma. ^a^Split at median; ^b^ *P*-value from Chi-square or Fisher exact test; ^*^Statistically significant (*P* < 0.05).

### Prognostic significance of FGL2 in patients with ccRCC

**Figure 3:**
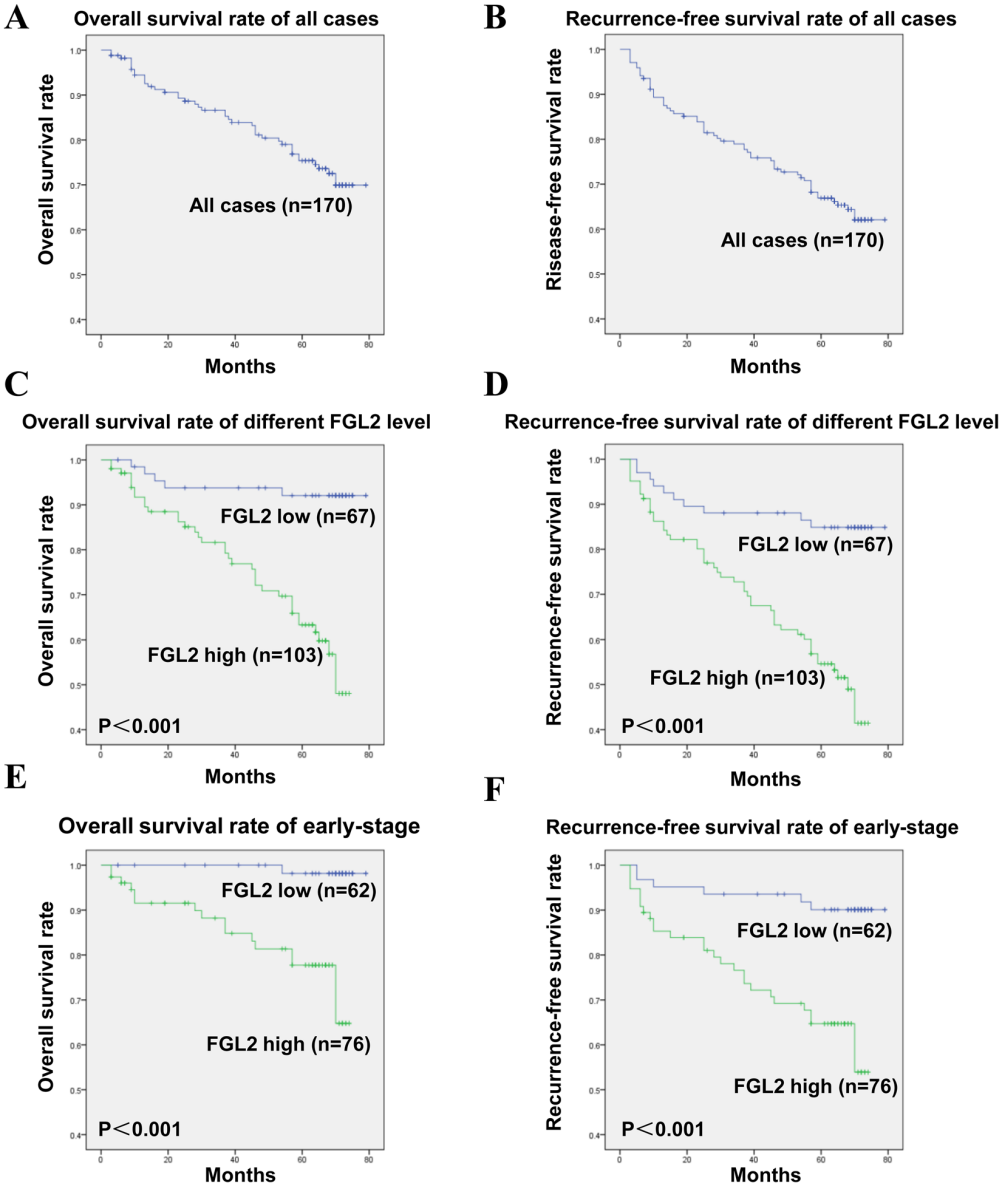
Analysis of overall survival (OS) and recurrence-free survival (RFS) of patients with ccRCC based on FGL2 expression by the Kaplan–Meier survival curve and log-rank test. (**A,B**) The five-year OS and RFS rate. (**C,D**) The patients with high FGL2 expression had significantly shorter OS and RFS than those with low FGL2 expression. (E,F) OS and RFS of all ccRCC patients in early-stage ccRCC according to tumoural FGL2 expression. Log rank test, *P* < 0.05 was regarded as statistically significant.

Five-year OS and RFS rates of 170 patients with ccRCC were 75% and 67%, respectively (Fig. 3A,B). Kaplan-Meier analysis and log-rank test were used to assess whether different FGL2 expression levels significantly predicted the clinical outcomes of patients with ccRCC. Results of these analyses showed that patients with ccRCC who showed high FGL2 expression had significantly poorer OS (log-rank test: *P* < 0.001) and RFS (log-rank test: *P* < 0.001) than patients showing low FGL2 expression (Fig. 3C,D). To further evaluate the prognostic importance of FGL2 expression, we stratified the patients according to their clinical TMN stage, which is the most commonly used method in clinical practice, and re-performed survival analysis. We found that high FGL2 expression was associated with significantly poorer OS and RFS than low FGL2 expression in patients with early-stage ccRCC (TNM stage, I + II; log-rank test, *P* < 0.001; Fig. 3E,F). However, no significant correlation was observed between FGL2 expression and OS and RFS of patients with advanced-stage ccRCC (TNM stage, III + IV; log-rank test: P > 0.05) because of their small sample size (Fig. S2A,B). These results indicate that FGL2 is a vital prognostic biomarker for patients with ccRCC at least in the early stages of the disease. Univariate and multivariate analyses were performed to identify the correlation between FGL2 expression and postoperative survival. Univariate Cox regression analysis showed that tumour size, T classification, TNM staging, necrosis, and high FGL2 expression were independent predictors of OS (HR, 6.636; 95% CI, 2.579–17.080; *P* < 0.001; Table 2). Moreover, tumour size, T classification, TNM staging, Fuhrman grade, and high FGL2 expression were significantly associated with RFS (HR, 4.214; 95% CI, 2.119–8.383; *P* < 0.001). As expected, multivariate analysis based on Cox proportional hazards model showed that high FGL2 expression was an independent predictor of both OS and RFS (OS: HR, 3.396; 95% CI, 1.187–9.722; *P* = 0.023; RFS: HR, 2.940; 95% CI, 1.402–6.166; *P* = 0.004), like tumour size (*P* < 0.001), TNM staging (*P* = 0.001), Fuhrman grade (*P* < 0.001), and necrosis (*P* = 0.003). Together, these results indicate that FGL2 expression is an independent prognostic factor of OS and RFS in patients with ccRCC.

**Table 2:** Univariate and multivariate Cox regression analysis of different prognostic variables for overall survival (OS) and recurrence free survival (RFS) prediction in ccRCC patients.

Characteristic	OS				RFS			
	Univariate		Multivariate		Univariate		Multivariate	
	HR (95% CI)	*P* ^a^	HR (95% CI)	*P* ^a^	HR (95% CI)	*P* ^a^	HR (95% CI)	*P* ^a^
Gender(Male *vs* Female)	1.037(0.556–1.934)	0.908	1.205(0.615–2.358)	0.587	1.228(0.733–2.059)	0.435	1.343(0.782–2.307)	0.284
Age,years (>55 *vs* ≤55)	1.250(0.682–2.290)	0.471	0.999(0.496–2.013)	0.998	1.417(0.850–2.361)	0.181	1.274(0.725–2.238)	0.400
Tumor size,cm (>4 *vs* ≤4)	7.791(3.447–17.607)	<0.001*	4.596(1.600–13.198)	0.005*	7.472(3.773–14.797)	<0.001*	5.666(2.517–12.755)	<0.001*
T classification (T3 + T4 *vs* T1 + T2)	11.583(5.912–22.693)	<0.001*	0.753(0.167–3.394)	0.711	5.185(3.067–8.766)	<0.001*	0.337(0.094–1.209)	0.095
N classification (N1 *vs* N0)	2.099(0.646–6.818)	0.217	1.075(0.289–4.006)	0.914	1.904(0.688–2.267)	0.215	1.328(0.438–4.028)	0.616
TNM stage (III + IV *vs* I + II)	10.006(5.261–19.028)	<0.001*	9.571(2.034–45.026)	0.004*	5.157(3.025–8.793)	<0.001*	9.613(2.456–37.623)	0.001*
Fuhrman grade (3 + 4 *vs* 1 + 2)	1.742(0.916–3.316)	0.091	3.779(1.645–8.680)	0.002*	2.628(1.568–4.405)	<0.001*	4.978(2.669–9.282)	<0.001*
Necrosis(Present *vs* Absent)	2.493(1.153–5.392)	0.020*	4.808(1.649–14.017)	0.004*	1.905(0.936–3.876)	0.076	3.734(1.548–9.004)	0.003*
FGL2 expression (High *vs* low)	6.636(2.579–17.080)	<0.001*	3.396(1.187–9.722)	0.023*	4.214(2.119–8.383)	<0.001*	2.940(1.402–6.166)	0.004*

HR = hazard ratio; CI = confidence interval. ^a^ *P*-value from the Cox proportional hazards model; *Statistically significant (*P* < 0.05).

### Prognostic nomogram establishment for predicting the OS and RFS of patients with ccRCC

**Figure 4:**
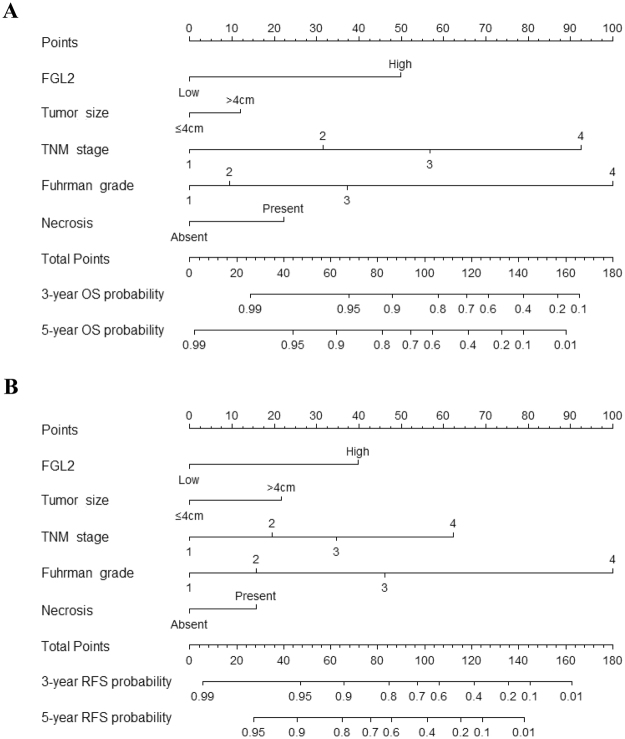
Nomogram for predicting 3-year and 5-year prognosis of ccRCC patients. (**A**) Nomogram for OS prediction. (**B**) Nomogram for RFS prediction.

**Figure 5:**
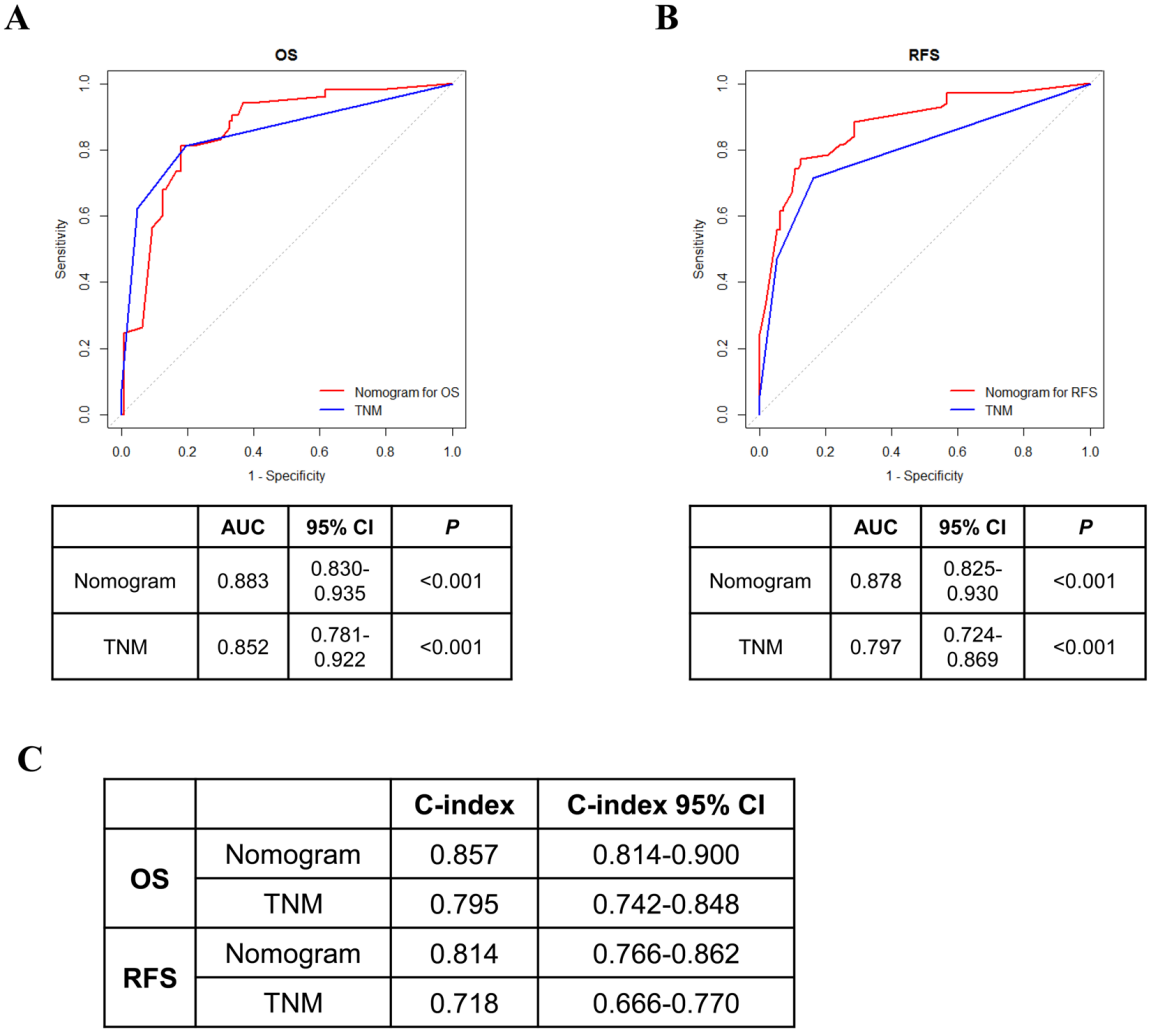
Comparison of the established nomogram model with TNM staging system. (**A** and **B**) ROC analyses for the sensitivity and specificity for the prediction of OS and RFS. *P*-value, calculated by z test, <0.05 was regarded as statistically significant. (**C**) Comparison of C-index. C-index = concordance index.

We integrated the significant prognostic factors determined by performing multivariate Cox regression analysis into a prediction system to establish two novel nomogram systems for predicting OS and RFS, respectively. These novel nomogram systems showed that FGL2 expression was a significant negative indicator of OS and RFS (Fig. 4A,B). ROS analyses for OS and RFS showed that the area under the curve (AUC) of the nomogram system was larger (OS: AUC, 0.883; 95% CI, 0.830–0.935; RFS: AUC, 0.878; 95% CI, 0.825–0.930) than that of the TNM staging system (OS: AUC, 0.852; 95% CI, 0.781–0.922; RFS: AUC, 0.797; 95% CI, 0.724–0.869) (Fig. 5A,B). Next, concordance index (c-index) of the novel nomogram system was compared with that of the TNM staging system. The integrated nomogram system had a higher c-index value than the TNM staging system (nomogram system and TMN staging system: c-index values of 0.857 and 0.795, respectively, for OS and of 0.814 and 0.718, respectively, for RFS). These results suggest that the novel integrated nomogram system is more accurate than the traditional TNM staging system for predicting the OS and RFS of patients with ccRCC.

### The correlation of FGL2 expression with ccRCC cells viability, apoptosis and ERK1/2 and p38 mitogen-activated protein kinase (MAPK) pathway activation

**Figure 6:**
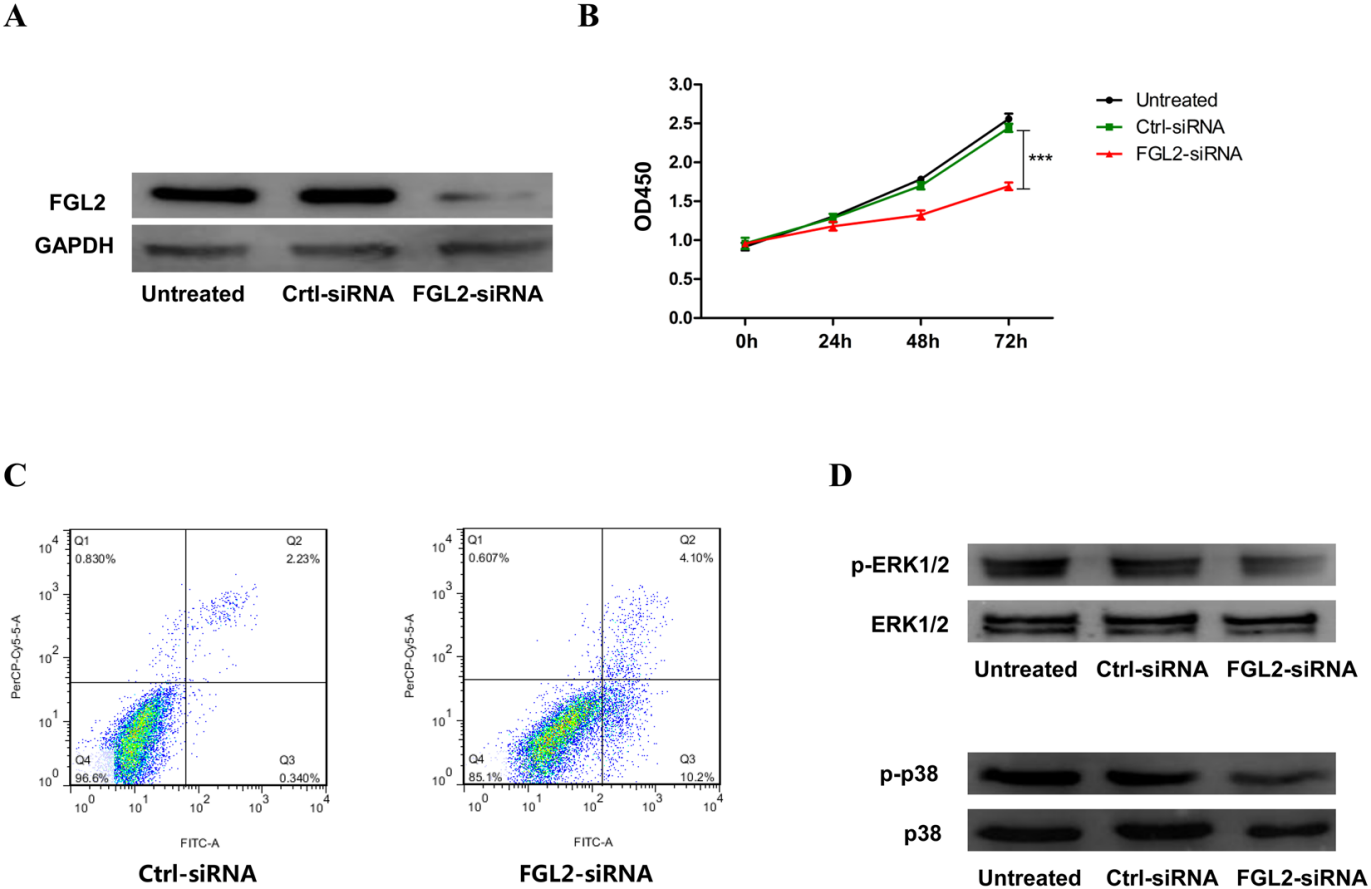
The biological role of FGL2 in ccRCC cells. (**A**) The levels of FGL2 protein in 786-O cells were detected by Western blotting analysis. (**B**) 786-O cells viability and (**C**) apoptosis in different groups were measured by CCK-8 kit and flowcytometry analysis. (**D**) Representative Western blotting of phosphorylated and total ERK1/2 and p38 MAPK protein in cultured 786-O cells. The blots were cropped and full-length blots were presented in Fig. S3A,B.

**Figure 7:**
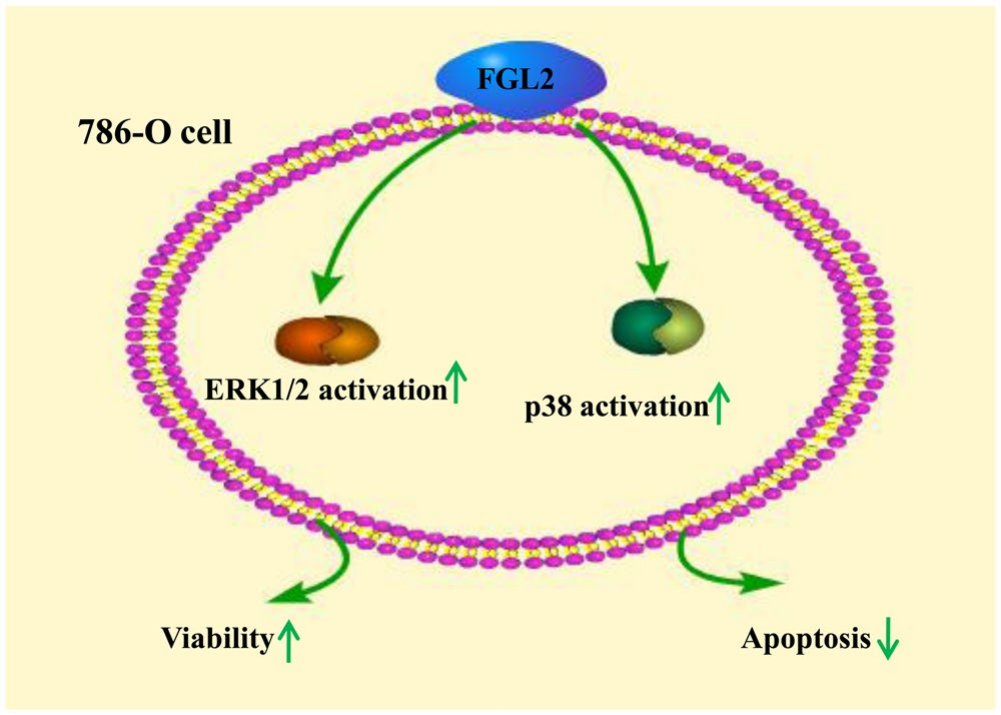
Schematic diagram depicts the potential mechanism of the role of FGL2 in ccRCC cells. FGL2 expression results in the increase in ccRCC cells viability and the reduction in ccRCC cells apoptosis by potentiating ERK1/2 and p38 MAPK pathway activation.

In order to further investigate the underlying mechanism of FGL2 in promoting ccRCC, we silenced FGL2 expression in ccRCC cell line 786-O cells by small interfering RNA (siRNA) *in vitro* (Fig. 6A). It was found that the silencing of FGL2 expression led to a significant reduction in cells viability and an increase in cells apoptosis, accompanied with a reduced activation in ERK1/2 and p38 MAPK pathway, an important signalling pathway in ccRCC^15,16^, in ccRCC cells (Fig. 6B–D). As shown in Fig. 7, the schematic diagram depicts the potential mechanism of the role of FGL2 in ccRCC cells.

## Discussion

Increased FGL2 expression has been detected in several human tumours, including lymphomas^14^, gliomas^17^, and hepatocellular carcinomas^18^. To our knowledge, the present study is the first to show an association between high FGL2 expression and unfavourable prognosis of patients with ccRCC after surgery. In the present study, we focused on FGL2 expression in ccRCC samples and found that FGL2 expression was significantly increased in tumour tissues compared with that in peritumoural tissues. We also found that high FGL2 expression was positively correlated with the stage of ccRCC. Further, we integrated tumoural FGL2 expression and other clinical parameters to generate two novel nomogram systems for predicting the RFS and OS of patients with ccRCC. Comparison by using ROC analyses and c-indexes showed that the novel nomogram systems were more accurate than the traditional TMN staging system for predicting the RFS and OS of patients with ccRCC. *In vitro* study, we demonstrated that FGL2 expression was related to ccRCC cells viability and ERK1/2 and p38 MAPK pathway activation.

FGL2 is expressed by activated endothelial cells and macrophages and is released by CD4^+^ and CD8^+^ T cells. As an immunoregulatory factor, FGL2 enhances regulatory T cell activation, balances Th1 and Th2 ratio, and suppresses antigen presentation activity to affect adaptive immunity^19–21^. FGL2 expression increases in PBMCs of patients with active B-cell lymphomas but normalizes after remission^14^. FGL2 induces CD39 expression in the tumour microenvironment^17^, which promotes the conversion of M1 macrophages to tumour-promoting M2 macrophages^22^. Furthermore, the mRNA information from 2013 TCGA cohort data included in the present study suggested that FGL2 expression was upregulated in ccRCC tissues and indicated that FGL2 expression was closely associated with the expression of tumour-promoting factors such as IL17 and IL-10 in ccRCC (Fig. S4A,B). This suggested that aberrant FGL2 expression in ccRCC interferes with tumour progression.

The MAPK cascade is a critical pathway for human cancer cells proliferation, growth, and division and crosses different malignancies^23,24^. It has well established that ERK1/2 and p38 MAPK pathway plays a significant role in ccRCC^15^. Moreover, it has been reported that FGL2 is associated with MAPK dependent cell proliferation and apoptosis^25^, and overexpression of FGL2 induces phosphorylation of p38 MAPK and ERK1/2 in HCCLM6 cells^13^. Consistent with these observations, at present study we found that *FGL2* silencing significantly inhibited ccRCC cells viability and ERK1/2 and p38 activation, and promoted cells apoptosis, suggesting that FGL2 aggravates the pathogenesis of ccRCC by promoting the activation of ERK1/2 and p38 MAPK signalling pathway.

RCC activates coagulation/fibrinolysis systems, and coagulation pathway plays an important role in RCC pathogenesis^26^. Tumour-related coagulation pathway is mediated by angiogenesis and by factors expressed on cancer cells, such as fibrinogen and coagulation factor VII^27–29^. Increased plasma fibrinogen levels are associated with poor histopathological and unfavourable survival outcomes, suggesting that fibrinogen levels can be used as a prognostic biomarker for patients with RCC^27^. As a fibrinogen-like protein, FGL2 contributes to renal ischemia by activating renal microthrombosis in patients with type 2 diabetic nephropathy^30^. FGL2 is highly expressed in HCC tissues and contributes to HCC angiogenesis and hypercoagulability^31^. Moreover, *FGL2* knockdown delays HCC growth and tumour angiogenesis^12,13^. Therefore, we speculate that FGL2 expression is highly associated with renal microthrombosis. Interestingly, FGL2 was highly expressed (staining index score, ≥ 6) in all patients with renal microthrombosis in the present study. However, these patients were not included in statistical analyses because of their small sample size (n = 7).

Although we determined the clinical significance of FGL2 expression in patients with ccRCC, our research has some limitations. First, this is a retrospective study, which is associated with inherent shortcomings. Second, this study has statistical limitations because of the inclusion of small number of patients, especially patients with advanced and metastatic ccRCC, indicating the need for a large, multicentre study. Third, an independent cohort is necessary to confirm the findings of the present study. Moreover, the association between high FGL2 expression and renal microthrombosis in ccRCC should to be investigated in further studies; Finally, additional *in vivo* studies are needed to further investigate the mechanisms of FGL2 in ccRCC.

In summary, we found that FGL2 expression level was significantly increased in ccRCC tissues. High FGL2 expression was an independent prognostic factor of poor OS and RFS in patients with ccRCC, and it is better performed in especially those with early-TNM-stage ccRCC. Integrating FGL2 expression with other clinical parameters in a nomogram surveillance system may improve the accuracy of predicting the OS and RFS of patients with ccRCC after surgery. FGL2 is expected to be a potential marker for prognosis and one possible target for therapy in ccRCC.

## Methods

### Tissue specimens

Fresh ccRCC tissues were collected from patients who underwent partial or radical nephrectomy at the Southwest Hospital of the Third Military Medical University (Chongqing, China) during 2016. In all, 170 paraffin-embedded tissue samples with pathologically validated ccRCC were collected between 2010 and 2011. Patients who did not have other malignant history and who did not undergo anticancer therapy were included. Exclusion criteria were (a) incomplete follow-up data, (b) tumour necrosis area of > 80%, and (c) death within the first month after surgery. Tumour stages were histologically classified according to the TNM classification of American Joint Committee on Cancer (2010)^32^. All the patients were followed up from the date of diagnosis to death or to the last follow-up date. Informed consent was obtained from all patients included in this study, and the study was approved by the ethical committee of the First Affiliated Hospital of Third Military Medical University (Southwest Hospital). All experimental protocols were carried out in “accordance” with the approved guideline.

### qRT-PCR

Total RNA was extracted from the tissues by using TRIzol reagent (Takara, Japan), according to the manufacturer’s protocol. First-strand cDNA was synthesized using a reverse transcription system (Takara, Japan), according to the manufacturer’s instruction, and mRNA levels were normalized to GAPDH. Primer sequences for PCR amplification are as follows: *FGL2* forward, 5′-AGGCAGAAACGGACTGTTGT-3′; *FGL2* reverse, 5′-CCAGGCGACCATGAAGTACA-3′; *GAPDH* forward, 5′-CTCTGCTCCTCCTGTTCGAC-3′ and *GAPDH* reverse, 5′-GCGCCCAATACGACCAAATC-3′. All samples were measured in triplicate. Differences in gene expression were calculated using 2^−ΔΔct^ method^33^.

### Western blotting

Total proteins were isolated from the renal tissues and 786-O cells by using RIPA buffer, and protein concentrations were quantified using BCA Protein Assay Kit (Beyotime, Shanghai, China). Protein samples (35 μg/lane) were resolved by performing sodium dodecyl sulphate-polyacrylamide gel electrophoresis and were transferred onto polyvinylidene difluoride membranes (Beyotime). The membranes were incubated overnight at 4 °C with mouse anti-human FGL2 antibody (Ab) (dilution, 1:400; Abnova, Taiwan), rabbit anti-human GAPDH antibody (Ab) (dilution, 1:1000; Abcam, Cambridge, MA, USA), rabbit anti-human ERK1/2 antibody, mouse anti-human phospho-ERK1/2 antibody, rabbit anti-human p38 antibody or phospho-p38 antibody (all dilution, 1:1000; Beyotime, Shanghai, China) followed by incubation with horseradish peroxidase-conjugated goat anti-mouse IgG or goat anti-rabbit IgG secondary Abs (dilution, 1:3000; ZSGB-BIO, Beijing, China). Immunoblots were visualized using a ECL Western Blotting Detection System (Millipore, Billerica, MA, USA). GAPDH was used as a loading control.

### Immunohistochemical staining and assessment

Primary mouse anti-FGL2 Ab (dilution, 1:200; Abnova, Taiwan) was used for immunohistochemical analysis as described previously^34^. The intensity of immunostaining was examined and scored by two independent pathologists who were blinded to the clinicopathological data. A semi-quantitative staining index ranging from 0 to 12 was calculated by multiplying staining intensities (0: negative, 1: weak, 2: moderate, and 3: strong) with the proportion of positively stained tumour cells (0: <5% positive cells; 1: 5–25% positive cells; 2: 26–50% positive cells; 3: 51–75% positive cells; and 4: >75% positive cells) for each sample. Staining index scores of ≥6 and ≤4 indicated high and low FGL2 expression, respectively.

### Transfections with FGL2 small interfering RNA

786-O cells were plated at 1 × 10^3^ cells/cm^2^ in complete medium without antibiotics. After 60% confluent, cells were transfected with 10 μM siRNA for FGL2 and scramble control siRNA (Santa Cruz Biotechnology) using Lipofectamine 2000 (Invitrogen) according to the manufacturer’s protocol. At 6 hours later, the medium was replaced with complete medium for 24 hours. To confirm the efficacy of the siRNA on the expression of FGL2, protein was isolated and assayed by Western blotting.

### Cell proliferation and apoptosis assays

Cell proliferation was measured using commercially available CCK-8 kits (Dojinodo, Shanghai, China) according to the manufacturer’s instructions. Cell apoptosis was measured by using flow cytometry as previously described^35^.

### Statistical analysis

Data were analysed using SPSS 19.0 (SPSS Inc., IL, Chicago, USA), GraphPad Prism 6 (GraphPad Software Inc., La Jolla, CA, USA), and R software 3.3.2 with “rms” package (R Foundation for Statistical Computing, Vienna, Austria). Mann–Whitney test was used to compare the immunohistochemical staining index scores of tumoural and peritumoural tissues. The relationship between FGL2 expression and clinicopathological parameters of patients with ccRCC was evaluated using Chi-square test and Fisher’s exact test, as appropriate. Survival curves of OS and RFS were illustrated using Kaplan–Meier analyses and log-rank tests. Univariate and multivariate Cox proportional hazard models were used to evaluate HR and 95% CI. Two nomograms were constructed by integrating parameters showing statistical significance in Cox univariate analysis and FGL2 expression to predict the RFS and OS of patients with ccRCC. Predictive accuracy and sufficiency of the different models was evaluated using ROC analyses and Harrell’s concordance index (c-index). *P* < 0.05 was considered statistically significant.

### Data availability

All datasets generated or analyzed during this study are included in this published article.

## Electronic supplementary material

Supplementary Information

## References

[1] Zhang D (2017). PGRMC1 Is a Novel Potential Tumor Biomarker of Human Renal Cell Carcinoma Based on Quantitative Proteomic and Integrative Biological Assessments. PloS one.

[2] Bedke, J. *et al*. Systemic therapy in metastatic renal cell carcinoma. *World journal of urology*, doi:10.1007/s00345-016-1868-5 (2016). 10.1007/s00345-016-1868-5 PMC5272893 27277600

[3] Weiss RH, Lin PY (2006). Kidney cancer: identification of novel targets for therapy. Kidney Int.

[4] Rini BI, Campbell SC, Escudier B (2009). Renal cell carcinoma. Lancet.

[5] Sun M (2011). Prognostic factors and predictive models in renal cell carcinoma: a contemporary review. European urology.

[6] Frank I (2002). An outcome prediction model for patients with clear cell renal cell carcinoma treated with radical nephrectomy based on tumor stage, size, grade and necrosis: the SSIGN score. The Journal of urology.

[7] Shalev I (2010). The Role of FGL2 in the Pathogenesis and Treatment of Hepatitis C VirusInfection. Rambam Maimonides medical journal.

[8] Ning Q (2005). Role of fibrinogen-like protein 2 prothrombinase/fibroleukin in experimental and human allograft rejection. Journal of immunology.

[9] Hu J (2016). The Duality of Fgl2 - Secreted Immune Checkpoint Regulator Versus Membrane-Associated Procoagulant: Therapeutic Potential and Implications. International reviews of immunology.

[10] Levy GA (2000). Molecular and functional analysis of the human prothrombinase gene (HFGL2) and its role in viral hepatitis. The American journal of pathology.

[11] Rabizadeh E (2015). The cell-membrane prothrombinase, fibrinogen-like protein 2, promotes angiogenesis and tumor development. Thrombosis research.

[12] Wang M, Liu J, Xi D, Luo X, Ning Q (2016). Adenovirus-mediated artificial microRNA against human fibrinogen like protein 2 inhibits hepatocellular carcinoma growth. The journal of gene medicine.

[13] Liu Y (2012). Downregulation of FGL2/prothrombinase delays HCCLM6 xenograft tumour growth and decreases tumour angiogenesis. Liver International.

[14] Rabizadeh E (2014). Increased activity of cell membrane-associated prothrombinase, fibrinogen-like protein 2, in peripheral blood mononuclear cells of B-cell lymphoma patients. PloS one.

[15] Pengcheng, S. *et al*. MicroRNA-497 suppresses renal cell carcinoma by targeting VEGFR-2 in ACHN cells. *Bioscience reports* **37**, doi:10.1042/BSR20170270 (2017). 10.1042/BSR20170270 PMC5437937 28465356

[16] Miyazaki A, Miyake H, Fujisawa M (2016). *Molecular mechanism mediating cytotoxic activity of axitinib in sunitinib-resistant human renal cell carcinoma* cells. Clinical & translational oncology: official publication of the Federation of Spanish Oncology Societies and of the National Cancer Institute of Mexico.

[17] Yan, J. *et al*. FGL2 as a Multimodality Regulator of Tumor-Mediated Immune Suppression and Therapeutic Target in Gliomas. *Journal of the National Cancer Institute* **107**, doi:10.1093/jnci/djv137 (2015). 10.1093/jnci/djv137 PMC4554195 25971300

[18] Su K (2008). Fibrinogen-like protein 2/fibroleukin prothrombinase contributes to tumor hypercoagulability via IL-2 and IFN-gamma. World journal of gastroenterology.

[19] Shalev I (2009). The novel CD4 + CD25 + regulatory T cell effector molecule fibrinogen-like protein 2 contributes to the outcome of murine fulminant viral hepatitis. Hepatology.

[20] Chan CW (2003). Soluble fibrinogen-like protein 2/fibroleukin exhibits immunosuppressive properties: suppressing T cell proliferation and inhibiting maturation of bone marrow-derived dendritic cells. Journal of immunology.

[21] Foerster K (2010). The novel immunoregulatory molecule FGL2: a potential biomarker for severity of chronic hepatitis C virus infection. Journal of hepatology.

[22] Dwyer KM (2007). CD39 and control of cellular immune responses. Purinergic signalling.

[23] Zhou, A. Y. & Johnson, D. B. Combinatorial Therapies in Melanoma: MAPK Inhibitors and Beyond. *American journal of clinical dermatology*, doi:10.1007/s40257-017-0320-y (2017). 10.1007/s40257-017-0320-y PMC10184503 28861871

[24] Burotto M, Chiou VL, Lee JM, Kohn EC (2014). The MAPK pathway across different malignancies: a new perspective. Cancer.

[25] Zhenzhong, Z., Yafa, Y. & Jin, L. Fibrinogen-like protein 2 gene silencing inhibits cardiomyocytes apoptosis, improves heart function of streptozotocin-induced diabetes rats and the molecular mechanism involved. *Bioscience reports* **35**, doi:10.1042/BSR20150078 (2015). 10.1042/BSR20150078 PMC4613684 26182381

[26] Tsimafeyeu IV, Demidov LV, Madzhuga AV, Somonova OV, Yelizarova AL (2009). Hypercoagulability as a prognostic factor for survival in patients with metastatic renal cell carcinoma. Journal of experimental & clinical cancer research: CR.

[27] Erdem S (2014). Increased preoperative levels of plasma fibrinogen and D dimer in patients with renal cell carcinoma is associated with poor survival and adverse tumor characteristics. Urologic oncology.

[28] Rickles FR, Patierno S, Fernandez PM (2003). Tissue factor, thrombin, and cancer. Chest.

[29] Versteeg HH, Peppelenbosch MP, Spek CA (2003). Tissue factor signal transduction in angiogenesis. Carcinogenesis.

[30] Su G (2011). Fibrinogen-like protein 2 expression correlates with microthrombosis in rats with type 2 diabetic nephropathy. Journal of Biomedical Research.

[31] Su K (2008). Fibrinogen-like protein 2/fibroleukin prothrombinase contributes to tumor hypercoagulability via IL-2 and IFN-γ. World journal of gastroenterology.

[32] Kim SP (2011). Independent validation of the 2010 American Joint Committee on Cancer TNM classification for renal cell carcinoma: results from a large, single institution cohort. The Journal of urology.

[33] Arya M (2005). Basic principles of real-time quantitative PCR. Expert review of molecular diagnostics.

[34] Xu L (2014). Prognostic value of diametrically polarized tumor-associated macrophages in renal cell carcinoma. Annals of surgical oncology.

[35] Li J (2016). Downregulated miR-506 expression facilitates pancreatic cancer progression and chemoresistance via SPHK1/Akt/NF-kappaB signaling. Oncogene.

