# Supplementary material for: Increased expression of Fibrinogen-Like Protein 2 is associated with poor prognosis in patients with clear cell renal cell carcinoma
Source: Sci Rep. 2017 Oct 4;7:12676. doi: 10.1038/s41598-017-13149-x (PMC5627263; doi:10.1038/s41598-017-13149-x)
Supplement: Supplementary file 1 — Supplementary Information [file 41598_2017_13149_MOESM1_ESM.pdf]

Increased expression of Fibrinogen-Like Protein 2 is associated with poor prognosis in patients with clear cell renal cell carcinoma

Ming Tang[1,*], Xu Cao[1,*], Peng Li[1], Kun Zhang[1], You Li[1], Quan-you Zheng[1], Gui-qing Li[2], Jian Chen[2], Gui-lian Xu[2], Ke-qin Zhang[1]

[1]Department of Nephrology, Southwest Hospital, Third Military Medical University, Chongqing 400038, China. [2]Department of Immunology, Third Military Medical University, Chongqing 400038, China. [*]These authors contributed equally to this work. Correspondence should be addressed to K.Z. (E-mail: zhkq2004@163.com).

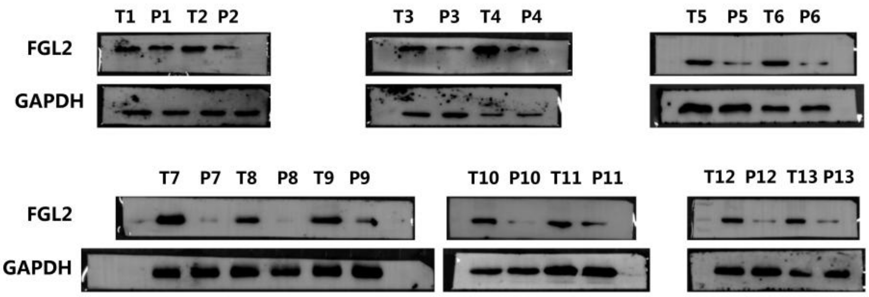

Figure S1. FGL2 overexpression in ccRCC specimens. The protein expression levels of FGL2 in fresh tumoural tissues and peritumoural tissues were evaluated by Western blotting assay. GAPDH was used as a loading control. P = Peritumour; T = Tumour.

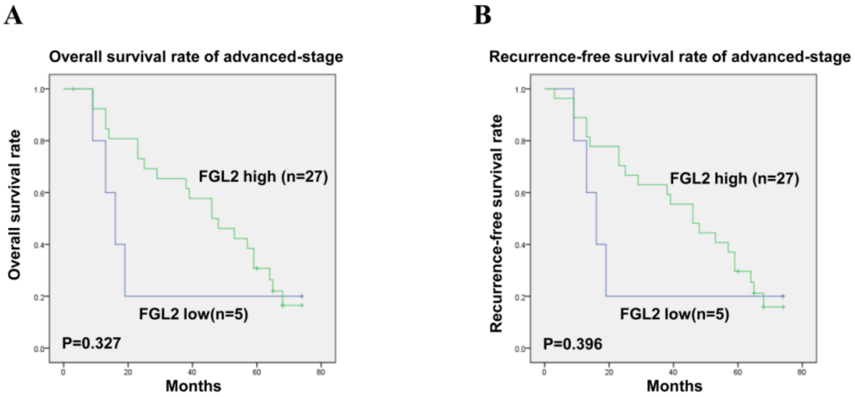

Figure S2. Analysis of overall survival (OS) and recurrence-free survival (RFS) of patients with advanced-stage ccRCC. (A) OS and (B) RFS of patients with advanced-stage ccRCC (TNM III+IV). according to FGL2 expression by the Kaplan-Meier survival curve and log-rank test.

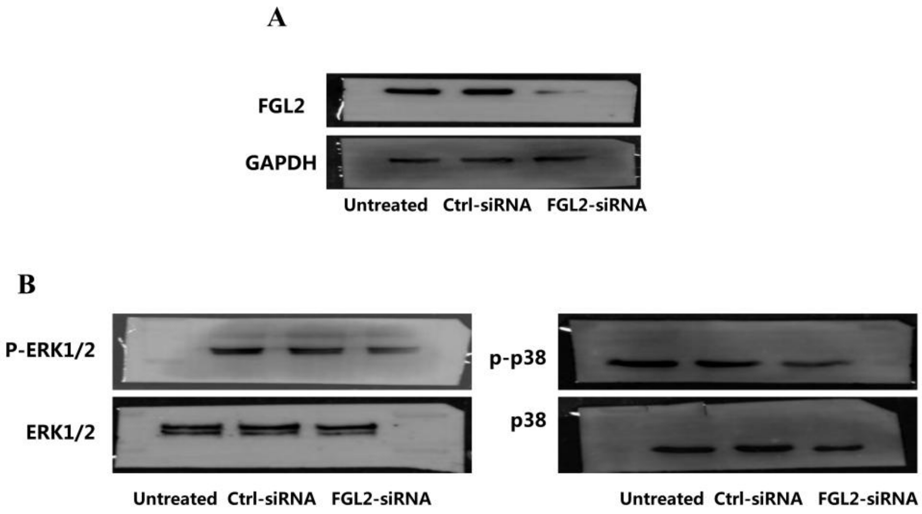

Figure S3. The biological role of FGL2 in 786-O cells. (A)The levels of FGL2 protein in 786-O cells were detected by Western blotting analysis and (B) representative Western blotting of phosphorylated and total ERK1/2 and p38 MAPK protein in cultured 786-O cells.

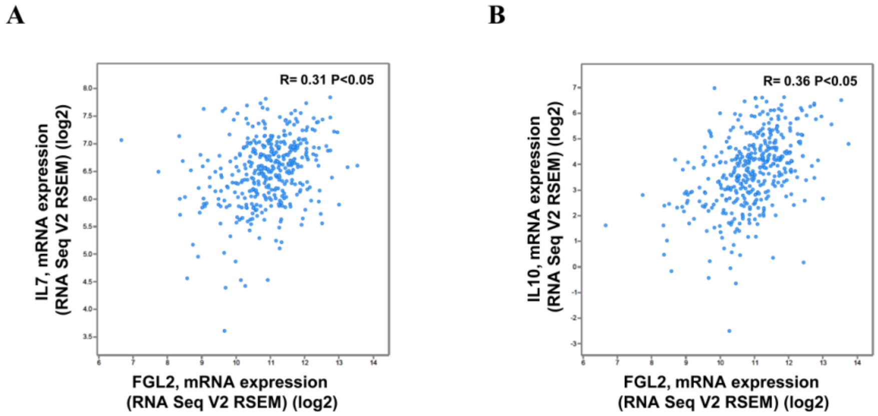

Figure S4. Correlation analysis of mRNA expression between FGL2 and tumour-promoting factors in patients with ccRCC. (A,B) Correlation between gene expression of the IL-17, IL-10 and FGL2 were analyzed by Pearson test. mRNA expression data of these genes from the TCGA database (2013). R = Pearson correlation coefficient. P <0.05 was regarded as statistically significant.

NO.	Gender	Age	Operation date	NO.	Gender	Age	Operation date
1	Female	54	2017/06/08	21	Female	30	2016/09/12
2	Male	59	2017/06/30	22	Male	54	2016/09/13
3	Female	30	2017/05/08	23	Female	65	2016/09/18
4	Male	42	2017/05/15	24	Male	37	2016/09/23
5	Male	50	2017/05/23	25	Male	42	2016/09/29
6	Female	66	2017/04/10	26	Male	51	2016/08/09
7	Female	52	2017/04/11	27	Male	44	2016/08/12
8	Male	53	2017/04/25	28	Female	45	2016/08/18
9	Male	52	2017/04/26	29	Female	56	2016/08/22
10	Female	55	2017/03/06	30	Male	42	2016/08/23
11	Male	69	2017/03/24	31	Female	44	2016/07/07
12	Male	68	2017/03/27	32	Male	49	2016/07/12
13	Male	76	2017/03/30	33	Female	50	2016/07/19
14	Male	56	2017/03/31	34	Female	66	2016/07/21
15	Female	52	2017/02/07	35	Male	32	2016/07/25
16	Male	43	2017/02/14	36	Male	46	2016/07/26
17	Male	57	2017/02/15	37	Female	42	2016/07/30
18	Female	56	2017/02/24	38	Female	61	2016/06/15
19	Female	47	2017/02/24	39	Female	51	2016/06/15
20	Male	77	2016/09/05				

Supplementary Table S1. Basic information of patients with ccRCC.
